# Zebrafish Otolith Biomineralization Requires Polyketide Synthase

**DOI:** 10.1016/j.mod.2019.04.001

**Published:** 2019-04-08

**Authors:** Kevin D. Thiessen, Steven J. Grzegorski, Yvonne Chin, Lisa Higuchi, Christopher J. Wilkinson, Jordan A. Shavit, Kenneth L. Kramer

**Affiliations:** 1 Department of Biomedical Sciences, Creighton University School of Medicine, Omaha, NE, United States; 2 Department of Pediatrics, University of Michigan, Ann Arbor, MI, United States; 3 Department of Biomedical Sciences, Royal Holloway University of London, London, England

**Keywords:** inner ear, otolith, biomineralization, calcium carbonate, polyketide synthase, zebrafish, endothelin-1, eNOS

## Abstract

Deflecting biomineralized crystals attached to vestibular hair cells are necessary for maintaining balance. Zebrafish (*Danio rerio*) are useful organisms to study these biomineralized crystals called otoliths, as many required genes are homologous to human otoconial development. We sought to identify and characterize the causative gene in a trio of homozygous recessive mutants, *no content* (*nco*) and *corkscrew* (*csr*), and *vanished* (*vns*), which fail to develop otoliths during early ear development. We show that *nco*, *csr*, and *vns* have potentially deleterious mutations in polyketide synthase (*pks1*), a multi-modular protein that has been previously implicated in biomineralization events in chordates and echinoderms. We found that Otoconin-90 (Oc90) expression within the otocyst is diffuse in *nco* and *csr*; therefore, it is not sufficient for otolith biomineralization in zebrafish. Similarly, normal localization of Otogelin, a protein required for otolith tethering in the otolithic membrane, is not sufficient for Oc90 attachment. Furthermore, eNOS signaling and Endothelin-1 signaling were the most up- and down-regulated pathways during otolith agenesis in *nco*, respectively. Our results demonstrate distinct processes for otolith nucleation and biomineralization in vertebrates and will be a starting point for models that are independent of Oc90-mediated seeding. This study will serve as a basis for investigating the role of eNOS signaling and Endothelin-1 signaling during otolith formation.

## 1. Introduction

Otoconia and otoliths act as a mass load that increase the sensitivity of mechanosensory hair cells to the effects of gravity and linear acceleration in mammals and fish, respectively. While the morphology of otoconia (“ear particles”) and otoliths (“ear stones”) differ, the initial formation of bio-crystals rely on many homologous proteins [1].

Zebrafish otoliths are primarily composed of calcium carbonate (CaCO_3_), in the form of aragonite, which accounts for ~99% of the total otolithic mass with the remainder consisting of proteins called otoconins [2, 3]. Further analysis of teleost otoliths has identified more than 380 protein components [4]. Based on the level of protein expression or changes in the rate of otolith growth, the polymorph of calcium carbonate crystals can change [1, 5]. For example, knockdown of Starmaker results in otoliths made of calcite rather than aragonite [6]. There are three pairs of otoliths in zebrafish, which include the sagittae, lapilli, and asterisci. While the lapillus and sagitta nucleate early in zebrafish development, the asteriscus does not form until 11–12 days in development [7]. The center of the otoliths contains a proteinaceous core that acts as a site for otolith nucleation and biomineralization. This matrix lays the foundation for further otolith growth, which is mediated by daily deposition of additional otoconins and calcium carbonate molecules [2]. Otolith nucleation occurs when the otolith precursor particles (OPPs) bind to the tips of the immotile kinocilia of tether cells within the otic vesicle [8, 9]. Subsequent studies have demonstrated that the critical period of otolith seeding and nucleation starts at 18–18.5 hpf (hours post fertilization) and ceases by 24 hpf [1, 8, 10–12].

In mammalian inner ear development, Otoconin-90 (Oc90; the major protein component of otoconia) is necessary for otoconial seeding and nucleation [13–15]. Oc90 can bind Otolin-1 (Otol1) to establish a protein-rich matrix that serves as a scaffold for subsequent deposition of calcium carbonate [16, 17]. Additionally, *in vitro* studies have suggested that Oc90 and Otol1 act synergistically to modulate otoconial crystal morphology [17]. While Oc90 is not the major protein component in zebrafish otoliths, it plays an important role in otolith seeding and early development as *oc90*-morphants do not develop otoliths [1, 18]. While additional gene mutations have been identified that lead to otolith agenesis in zebrafish [19–24], the genes responsible for several zebrafish otolith mutants have been undetermined.

In this study, we sought to identify and characterize the causative gene in a trio of zebrafish mutants, *no content* (*nco*) *corkscrew* (*csr*), and *vanished* (*vns*), which fail to develop otoliths during early inner ear development. We provide genetic evidence that the causative gene is polyketide synthase (*pks1*; currently *wu:fc01d11*), a candidate gene that was previously identified as a key factor of biomineralization in Japanese medaka (*Oryzias latipes*) and sea urchin (*Hemicentrotus pulcherrimus*) [25]. Furthermore, we offer potential signaling pathways for *pks1* function during inner ear development in the zebrafish.

## 2. Materials and Methods

### Husbandry and maintenance

All zebrafish were maintained in a temperature-controlled (28.5°C) and light-controlled (14h on/10h off) room per standardized conditions. *nco* strain (jj149) was generated by an ENU screen on the AB background and obtained from ZIRC (Eugene, OR, USA)[26]. *csr* was a spontaneous mutant generated in a *bre*-KO2/*ntl*-GFP line (AB background). *vns* was a spontaneous mutant generated in a AB/TL background. All protocols were approved by Creighton University and the University of Michigan Animal Care and Use Committees.

### Whole genome and RNA-sequencing

Mutant *nco* embryos and wild-type (WT) clutchmates were phenotyped and collected during the critical period of otolith nucleation and seeding (24 hours post fertilization, hpf) and the whole embryo lysates (n=50) were submitted for RNA sequencing. Analysis was completed using MMAPPR (Mutation Mapping Analysis Pipeline for Pooled RNA-seq) as previously described [24]. Whole genome sequencing of *csr* phenotypically-mutant embryos (n=150) was performed and analyzed using MegaMapper as previously described [27]. Common SNPs were removed by the Single Nucleotide Polymorphism Database (dbSNPs). Reference sequences for both experiments were mapped to Zv9. All sequencing was conducted at the University of Nebraska Medical Center Genomics Core Facility. Accession numbers for *nco* RNA-seq and *csr* genome sequencing will be provided during review.

### mRNA and plasmid DNA rescue

WT mRNA and *pks1*^L905P^ were synthesized using mMessage Machine from a clone provided by Dr. Hiroyuki Takeda (University of Tokyo), cleaned on an RNeasy column, and subsequently injected into single-cell *csr* and *nco* embryos. Naked plasmid of the medaka *pks1* clone was injected into *vns* embryos. Overall penetrance of otolith formation was determined in all three mutants. Site-directed mutagenesis (Agilent) was used to generate the mutant clone containing the causative mutation in *csr* (*pks1*^L905P^ in Japanese medaka; *pks1*^A911P^ in zebrafish). Primers used for site-directed mutagenesis were:

pks1_L905P_Forward: 5′-GATATGGCGTGATGTCCGGTGACAGGTTGAAGATC-3′
pks1_L905P_Reverse: 5′-ATCTTCAACCTGTCACCGGACATCACGCCATATC-3′

### Pathway analysis

Pathway analysis of *nco* was performed using Ingenuity Pathway Analysis (QIAGEN Inc., https://www.qiagenbioinformatics.com/products/ingenuity-pathway-analysis [28]. The Ensembl Gene IDs were assigned to each gene and uploaded to IPA. Cut-off for gene expression analysis was set at 0.75 RPKM. The calculated z-score indicates a pathway with genes exhibiting increased mRNA levels (positive) or decreased mRNA levels (negative). No change in mRNA levels results in a z-score of zero.

### Genotyping

*csr, nco, and vns* samples were PCR-amplified and submitted for Sanger sequencing using the following primers:

nco_Forward: 5′-GGGAGGATGCTTGTTGTTGG-3′
nco_Reverse: 5′-GTGGCCCAGAATAGGATCCA-3′
csr_Forward: 5′-AAGACGGGGACATGACTCAG-3′
csr_Reverse: 5′-TTCAACAAACAGTGCTCCGG-3′
vns_Forward: 5′-GCCATCATTGGAATTGGATG-3′
vns_Reverse: 5-GGTGTTCCAGTCCCATGAGC-3′

### RT-PCR

All RNA was extracted from Danio rerio wild-type embryos (A/B strain). After collecting embryos at the separate time-points, the samples were homogenised in lysis buffer from the Quick- RNA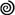 MiniPrep kit (Zymo Research-R1054) and RNA was extracted following protocol provided by the manufacturer. The RNA samples were then DNase treated using TURBO™ DNase (ThermoFisher, AM2238) as per manufacturer instructions, in order to remove any genomic contamination that may be present in the RNA. cDNA synthesis was achieved using the GoScript™ Reverse Transcription System (Promega, A5001) and followed the protocol provided by the manufacturer.

actb1_Forward: 5′-CTTCCAGCCTTCCTTCCT-3′
actb1_Reverse: 5′-CCACCGATCCAGACGGAGTA-3′
pks1_Forward: 5′-GAATTTTCTGCCGAGTAGAACAAAG-3′
pks1_Reverse: 5′-TCTGCATGTCAGGCGATCAG-3′

RT-PCR on the cDNA samples was carried out using the GoTaq® G2 Flexi DNA Polymerase (Promega, M7805) and PCR was done following the protocol provided by the manufacturer, using the primers stated above. The RT-PCR samples were then run on a 2% agarose gel.

### Immunofluorescence

*csr* and *nco* embryos were collected during key stages in early inner ear development, fixed with hydrogel and washed in CHAPS-based (1% by weight) CLARITY-clearing solution [29]. Embryos were decalcified with EDTA (120 mM in 0.1% PBS-Triton) before blocking (0.1% PBS-Triton with 3.33% sheep serum and 3.33% BSA), incubating in primary and secondary antibodies diluted in blocking buffer, mounting in 50% Glycerol-PBS solution, and imaging by confocal microscopy (Leica TCS SP8). Affinity-purified rabbit polyclonal antibodies were generated to Otogelin (CGNRVDGPSASKG; 1:1000) or Oc90 (CNTQSDTVDRKPTQSKPQ; 1:1000) by conventional methods (GenScript, USA) and directly labelled before immunofluorescence. Other antibodies used were Keratan Sulfate (MZ15; 1:2000; DSHB), Hair Cell Specific-1 (HCS-1; 1:500; DSHB), and acetylated-tubulin (1:500; Sigma T6793). Phalloidin (ThermoFisher A12379) was used at a concentration of 1:500.

### Mitotracker staining

Mitotracker Red (ThermoFisher #M22425) was resuspended in DMSO (0.25 mM) and diluted to 200 nM in E3 embryo medium. *nco* and *csr* embryos were then incubated in the dark for 20 minutes before removing Mitotracker solution and replacing with fresh E3 embryo medium. Samples were allowed to stabilize in the dark for 30 minutes before imaging at 21 hpf. Embryos were then phenotyped at 27 hpf.

### Exogenous salt solutions

To test the effects of exogenous ions on otolith formation, embryos were kept in E3 Medium until early gastrulation (~10 hpf). Embryos were washed, dechorionated, and transferred to 1X Basic Solution (58 mM NaCl, 0.4 mM MgSO_4_ and 5 mM HEPES) supplemented with 0.7 mM potassium chloride, 0.6 mM calcium nitrate or 0.6 mM calcium chloride. Embryos were then transferred to fresh 1X Basic Solution with respective supplement for the remaining development. Embryos were scored by the presence or absence of otoliths at 27 hpf and genotyped using High Resolution Melt analysis.

### Statistical analyses

Statistical significance was calculated using Fisher’s Exact Test, G-test for Independence, and Chi-Squared Distribution.

## 3. Results

### 3.1 csr and nco are genetically-linked

**Figure 1:**
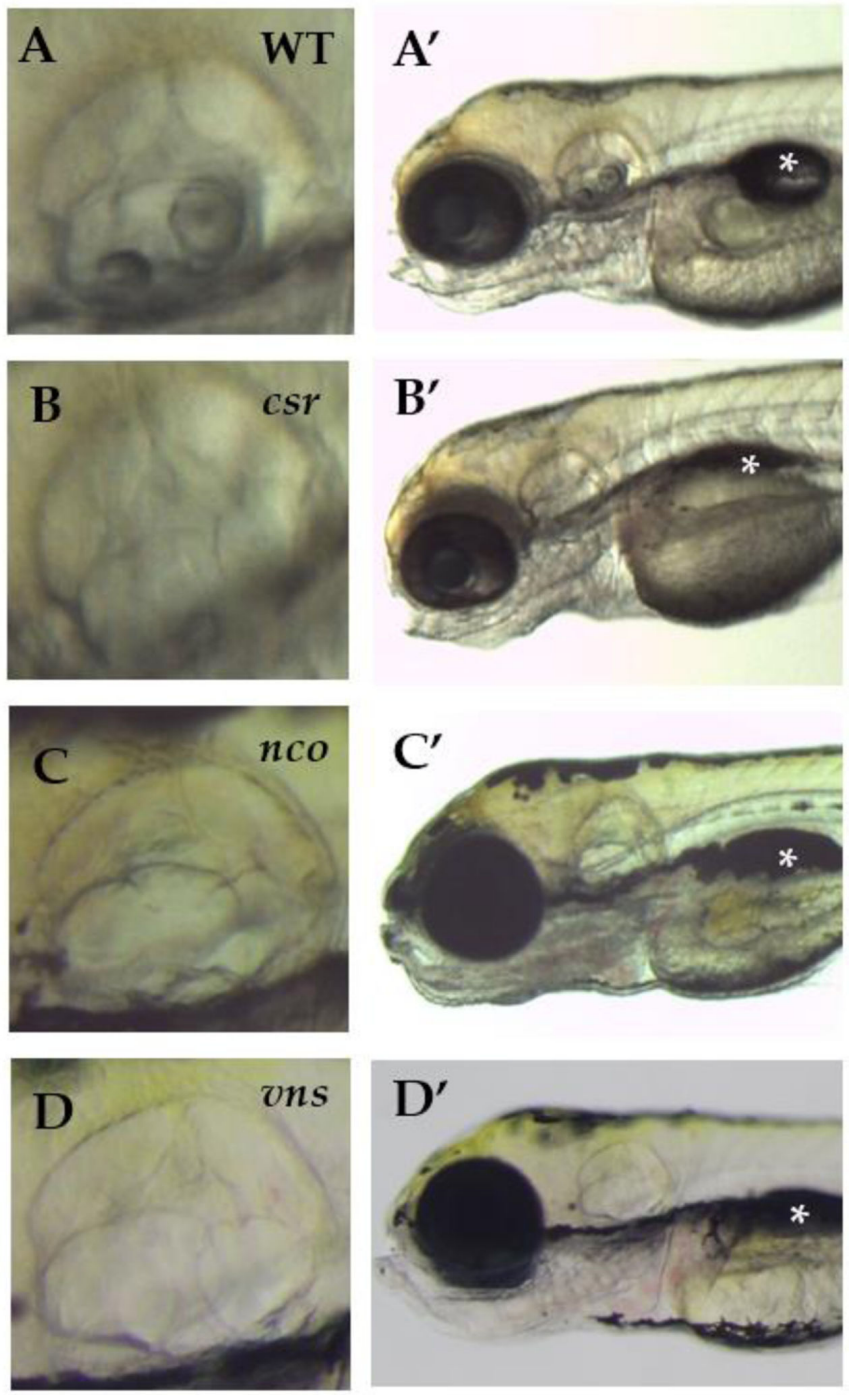
(**A-D**) The *csr*, *nco*, and *vns* mutant phenotypes fail to form otoliths within the inner ear. However, semicircular canal formation appears to be normal. (**A′-D′**) All mutants fail to inflate their swim bladders, which is lethal. Imaged at 5 days post fertilization (dpf). Magnification 6.3X. (*) indicates swim bladder.

The most apparent phenotype of the homozygous recessive *csr, nco*, and *vns* mutants is that they fail to form otoliths (lapillus and sagitta) or any observable complex calcium deposits within the inner ear (Fig. 1A–D; Table S1). Furthermore, the mutant larvae are homozygous lethal by 7 days post fertilization (dpf) as the swim bladder fails to inflate (Fig. 1A′–D′) and they are unable to feed. As a result, we do not know whether asteriscus formation is affected. While it is still unknown why the swim bladder fails to inflate when otoliths are absent, it is a common phenotype in other mutants with otolith agenesis [18–24]. Due to this commonality within *csr* and *nco*, we sought to determine if these phenotypes would complement each other. The results of the complementation test showed that some offspring failed to develop otoliths (29.25%; n=106; Table S1), supporting that *nco* and *csr* likely are allelic.

### 3.2 Exogenous ions influence otolith nucleation in csr embryos; not nco or vns embryos

As an aquatic species, the environment of zebrafish can be easily controlled and adapted to assess its impact on embryonic development. Previously, small molecules have been used to block otolith development by inhibiting otolith nucleation [10]. We hypothesized that there was an error in ion homeostasis that could be affected by exogenous solutions. In water treatments supplemented with calcium chloride (n=51), we found a significant decrease in *csr* penetrance in homozygous embryos (χ^2^=19.27, df=6; p=0.0037) compared to treatments supplemented with potassium chloride (n=46) or calcium nitrate (n=54). Additionally, we observed no significant change in *nco* mutant phenotype penetrance for water treatments supplemented with potassium chloride (17.76%; n=107), calcium chloride (16.67%; n=120) or calcium nitrate (16.9%; n=112)(G-test; p=0.975). Similarly, the penetrance of otolith formation in *vns* was not affected by exogenous salts (data not shown).

Building on the hypothesis that there was an error in ion homeostasis, Mitotracker was used to mark mitochondria-rich cells (i.e. presumptive ionocytes) in *csr* and *nco* embryos. While *nco* embryos appear normal, we observed that *csr* embryos show a lack of Mitotracker localization at 21 hpf (Fig. S1). Altogether, this suggests the nature of the *nco* and *csr* mutation, while likely allelic, are inherently different.

### 3.3 Potentially deleterious mutations identified in polyketide synthase for csr, nco, and vns

**Figure 2:**
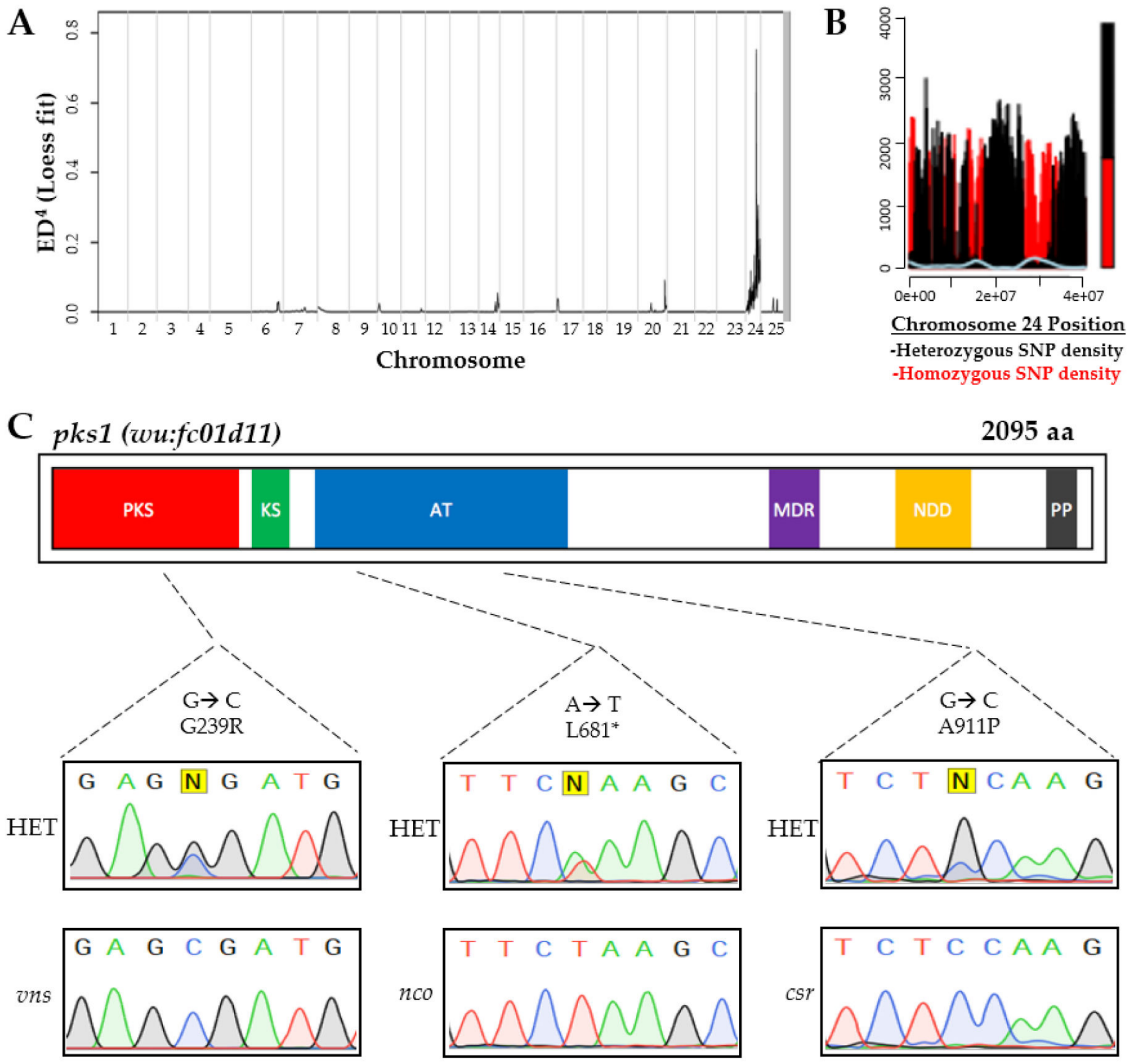
Complementary approaches for causative gene discovery. MMAPPR analysis of RNA sequencing data for *nco* (**A**) and whole genome homology mapping for *csr* (**B**) identified regions of high homology on the 24^th^ chormosome near the *pks1* locus (~33 Gb). (**C**) Deleterious mutations were identified in *pks1* for *nco* and *csr* within the acyl transferase (AT) domain and *vns* within the polyketide synthase (PKS) domain. Sanger sequencing confirmed SNPs in *csr*, *nco*, and *vns* mutants. Other domains include Ketoacyl Synthetase (KS), Medium Chain Reductase (MDR), NAD(P)-dependent dehydrogenase (NDD), and Phosphopanthetheine-Binding (PP).

To positionally clone the gene responsible for *nco* and *csr*, we used complementary approaches for each strain. MMAPPR analysis of *nco*-derived RNA sequencing (Fig. 2A) [24] and MegaMapper analysis of *csr-*derived whole genome sequencing (Fig. 2B) [27] both identified a genomic region with high homology surrounding the *pks1* locus. While several other genes were in that region, a previous study on otolith biomineralization in Japanese medaka made *pks1* the likely gene candidate [25]. Potentially deleterious mutations were identified in *pks1* for *csr* (A911P) and *nco* (L681*), which were both located within a conserved acyl transferase domain (Fig. 2C). Furthermore, a deleterious mutation in *vns* (G239R) was serendipitously found to be linked to a neighboring gene during a separate study. The deleterious point mutation was identified by Sanger sequencing of the *pks1* locus and confirmed by relatively high penetrance of otolith agenesis (95%).

### 3.4 Japanese medaka pks1 mRNA or plasmid DNA rescues otolith biomineralization in csr, nco, and vns

**Figure 3:**
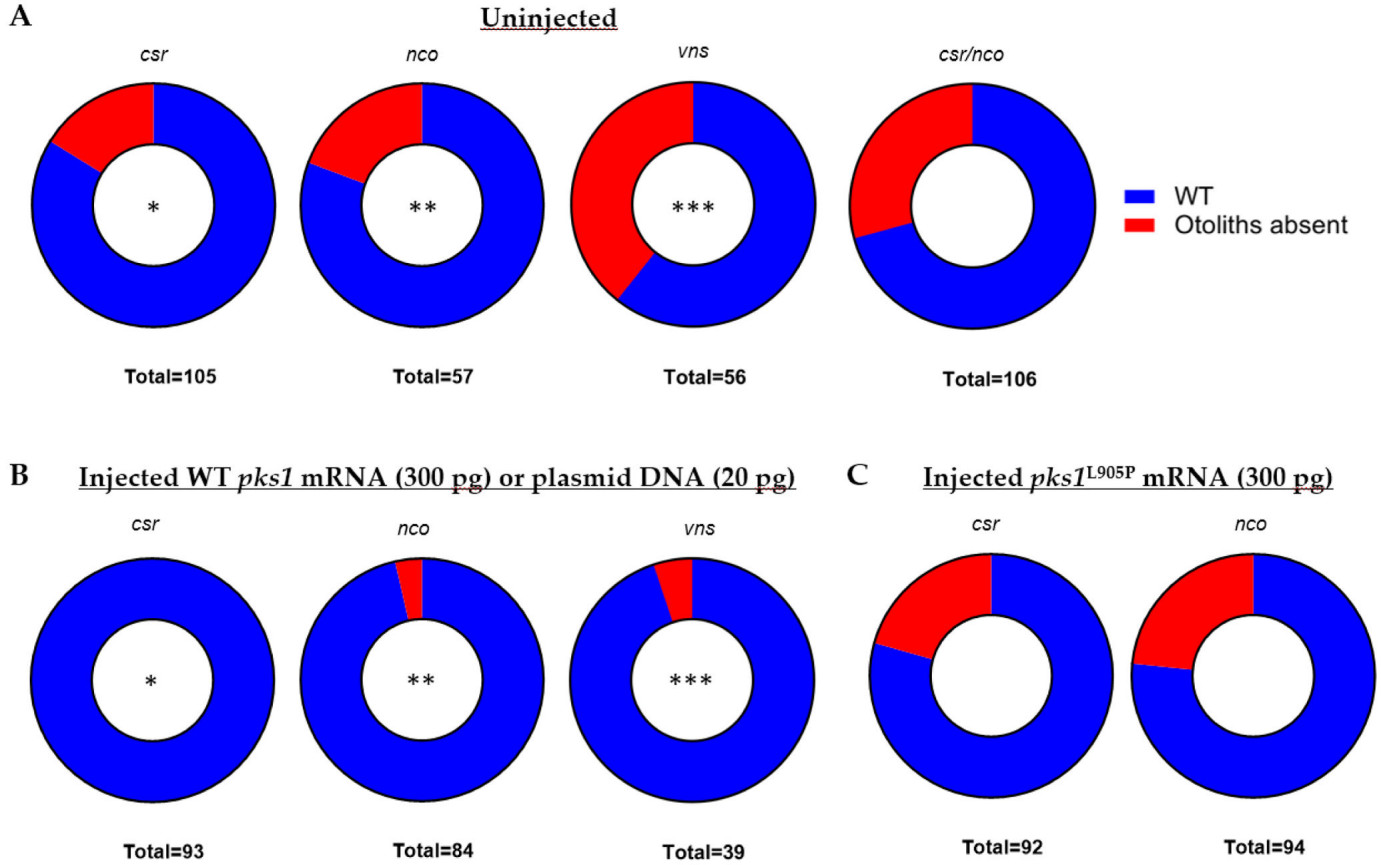
WT *pks1* nucleic acid rescues otolith formation in *csr*, *nco*, and *vns*. (**A**) Normal frequencies of mutant phenotypes in each uninjected strain. All four pairings follow homozygous recessive mode of inheritance. (**B**) Results of injected embryos show that Japanese medaka *pks1* mRNA (300 pg) rescues both *csr* and *nco* mutants and *pks1* DNA (20 pg) rescues *vns* mutants. (*, p < 0.0001, paired *t*-test)(^**^, p < 0.0032, paired *t*-test)(^***^, p = 0.0001, paired *t*-test),. Site-directed mutagenesis was used to introduce a conserved mutation in *csr* (A911P) into the Japanese medaka construct (L905P) (**C**) Injection of pks1^L905P^ (300 pg) fails to rescue *csr* or *nco* mutant phenotypes.

While the last common ancestor of Japanese medaka and zebrafish was estimated to be 150 million years ago [30], we sought to assess if the function of *pks1* within the inner ear is conserved. We injected Japanese medaka *pks1* mRNA or DNA into single-cell embryos of *csr, nco*, and *vns* heterozygous incrosses. Microinjection of Japanese medaka *pks1* mRNA (300 ng/μL) rescued otolith biomineralization in both *csr* (p<0.0001; *χ*^2^<0.0001; n=93) and *nco* (p=0.0032; *χ*^2^=0.0022; n=84) mutants (Fig. 3B; Table S1). Additionally, microinjection of the Japanese medaka *pks1* plasmid (20 ng/uL) provided by Dr. Takeda rescued otolith biomineralization in *vns* (p<0.0001; *χ*^2^=0.0004; n=39). Using site-directed mutagenesis, we introduced the non-synonymous mutation (A911P) in *csr* to the Japanese medaka mRNA construct (L905P). We repeated injections into single-cell embryos and failed to rescue otolith biomineralization in *csr* and *nco*. WT medaka *pks1*, but not *pks1*^*L905P*^, rescued otolith biomineralization in *csr* and *nco* embryos (Fig. 3C; Table S1).

### 3.5 Ingenuity pathway analysis of nco embryos

**Figure 4:**
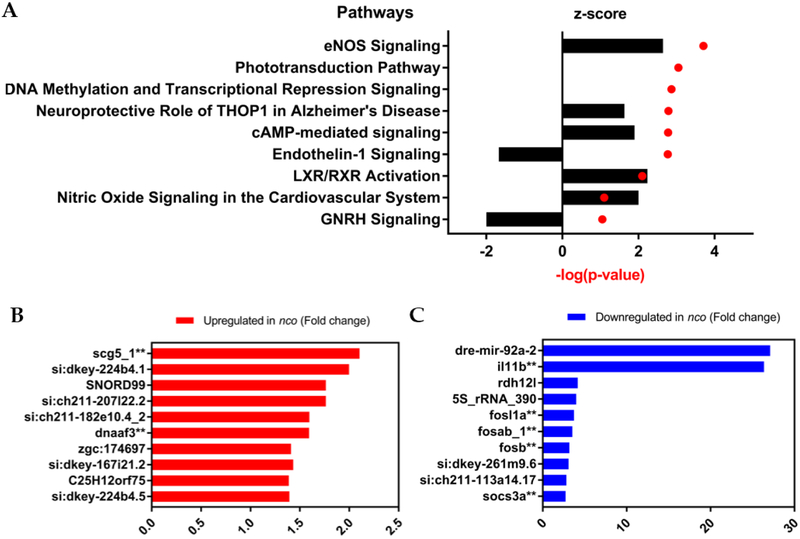
Gene expression and pathway analysis of *nco* embryos. (**A**) Ingenuity Pathway Analysis shows the top up-regulated and down-regulated pathways, which are eNOS Signaling and Endothelin-1 Signaling, respectively. Positive z-score indicated increased mRNA levels. Negative z-score indicates decreased mRNA levels. No change in mRNA levels results in a z-score of zero. (**B**) Differential gene expression in the top up-regulated genes. (**C**) Differential gene expression in the top down-regulated genes. (^**^, expressed in adult zebrafish mechanosensory hair cells) [32].

While *pks1* is thought to produce an otolith nucleation factor [25], its broader role during inner ear development is unknown. Ingenuity Pathway Analysis of *nco* at 24 hpf identified eNOS and Endothelin-1 signaling as the top up- and down-regulated pathways, respectively (Fig. 4A). Among the down regulated genes was *rdh12l*, a gene adjacent to *pks1*, suggesting that there is local control of transcription at that locus. *mir-92a*, the top down-regulated gene, has a predicted binding site in the 3′UTR of *rdh12l* (Fig. S2) [31]. In addition, several genes listed in the top ten up- or down-regulated lists are also enriched in adult mechanosensory hair cells such as *il11b, fosab, fosb, fosl1a, socs3a, scg5*, and *dnaaf3* (Figs. 4B–C) [32]. Of these genes, *il11b* is up-regulated during neuromast hair cell regeneration [33]. Notably, *dnaaf3* causes primary ciliary dyskinesia and morpholino knockdown of *dnaaf3* causes abnormal otolith growth [34]. While its role in inner ear development is unknown, *scg5* is expressed within the anterior and posterior poles of the otic placode during the critical period of otolith nucleation [35].

### 3.6 Aberrant expression of proteins involved in otolith development in csr and nco

**Figure 5:**
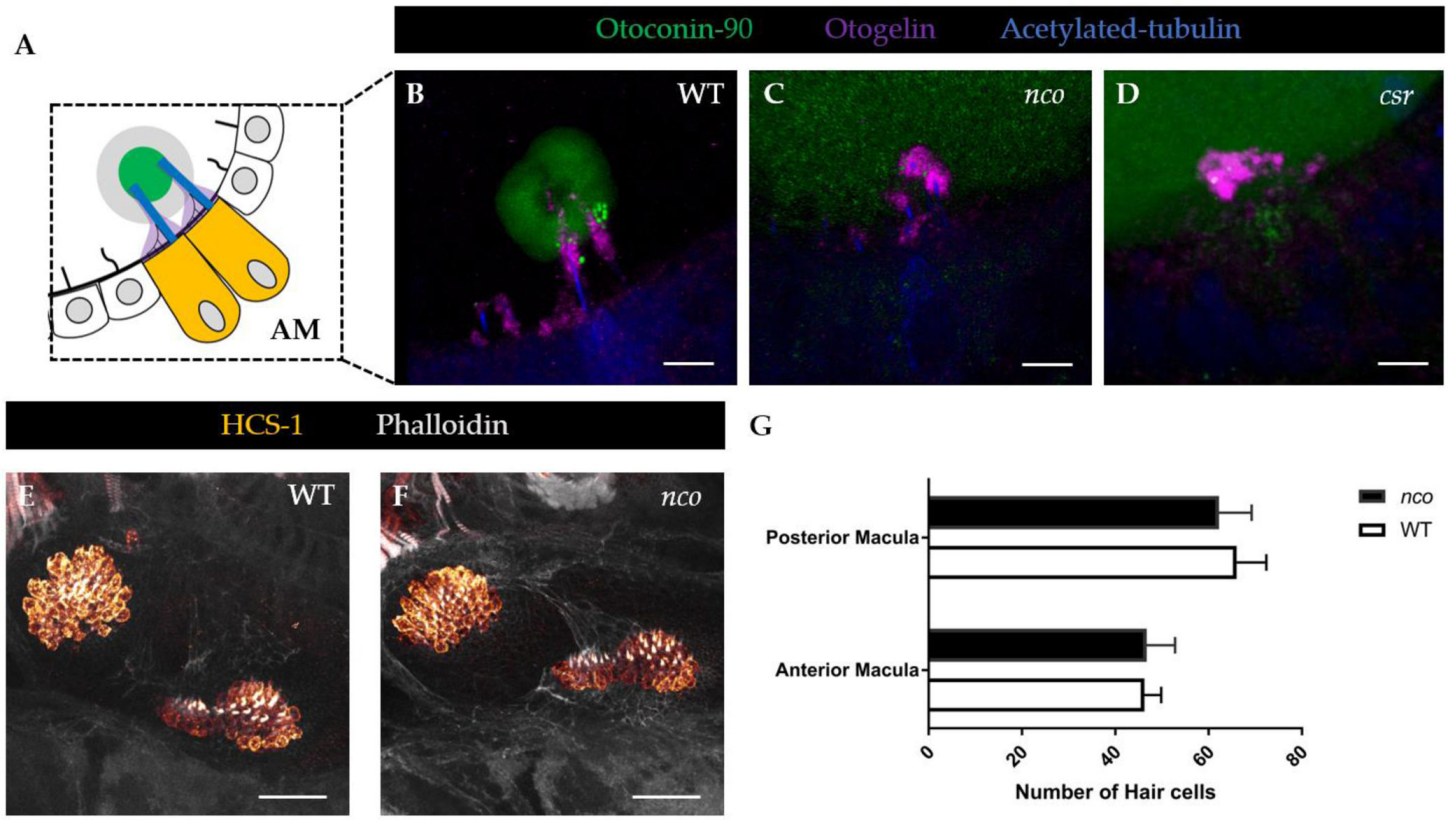
Aberrant expression of proteins invovled in otolith development in *csr* and *nco*. (**A**) Schematic of anterior macula (AM) tethered to otolith at 27 hpf. (**B**) In WT, Otoconin-90 (Oc90) is expressed within the mineralized otolith, which is situated atop the otolithic membrane (Otogelin, or Otog), at 27 hpf. Scale bar = 5 μm. (**C-D**) Oc90 has diffuse expression within the otocyst of *csr* and *nco*. In *csr* and *nco*, Otog is localized near the apical surface of hair cells. (**E-F**) Expression showing hair cells in WT and *nco* larvae at 5dpf. Scale bar = 25 μm. (**G**) Quantification of hair cell numbers in the posterior and anterior macula of WT and *nco* (n = 4).

In mammalian inner ear development, Oc90 is necessary for otoconial seeding and nucleation [13, 14]. Similarly, the role of Oc90 is evolutionarily-conserved in zebrafish and has been previously thought to be necessary for otolith nucleation [18]. Using immunofluorescence (IF), we saw diffuse expression of Oc90 in *csr* and *nco* otocysts (Figs. 5B–D), which demonstrated that Oc90 expression within the otocyst is not sufficient for otolith biomineralization in zebrafish. Similarly, normal localization of Otogelin (Otog), a protein required for otolith tethering in the otolithic membrane is not sufficient for Oc90 attachment. Additionally, other otoconins that are important for calcium deposition and growth were detected with diffuse expression within the otocyst such as Starmaker and Keratan Sulfate (data not shown) [36, 37].

### 3.7 Polyketide synthase as an otolith precursor binding factor?

Otolith nucleation is thought to be mediated by a tether-cell specific otolith precursor binding factor (OPBF), which lays the foundation for the successive biomineralization of the otolith [9, 11, 38]. The presence of an OPBF was proposed almost two decades ago and its identification proves to be elusive [38]. Recent studies suggest that one or more OPBFs are expressed by tether-cells and help to mediate otolith nucleation by binding other OPPs [9, 11, 39].

We sought to assess if *pks1* or its enzymatic product is a tether-cell specific nucleation factor. While medaka has diffuse pks1 mRNA expression in the otic epithelium [25], we hypothesized that the expression might be restricted to hair cells. First, using publicly available RNA-seq data, we found that *pks1* mRNA is enriched (7.46-fold increase) in adult mechanosensory hair cells compared to support cells within the zebrafish inner ear (Table S2). Additionally, this data suggests *pks1* mRNA to be transcriptionally regulated in support cells. Support cells predominantly express a 300bp region of the 5′UTR of the *pks1* transcript while hair cells express the full open reading frame [32]. A search for transcriptional regulatory motifs in the 5′UTR of *pks1* found a predicted binding site for TCF-3 [40], a transcription factor highly expressed in adult mechanosensory hair cells [32]. While the role of TCF-3 in the inner ear is unknown, it is expressed within the otic vesicle during the critical period of otolith nucleation [35].

Then, we demonstrated that the total number of hair cells remain unchanged during early development in *nco*, suggesting there are no differences in tether cell maturation and maintenance (Figs. 5E–G). Using RT-PCR, we detected *pks1* mRNA during the critical period of otolith nucleation (Fig. S3). However, *in situ* data showed ubiquitous expression of pks1 in the otic vesicle of zebrafish [25]. While *pks1* might be enriched in adult hair cells, early expression shows that it is ubiquitously expressed in the otic vesicle and, therefore, not the tether-cell specific OPBF.

## 4. Discussion

The homozygous recessive mutants *csr, nco*, and *vns* were chosen for this study because each lack the necessary factors such as an OPBF for otolith seeding and biomineralization. To determine the genes responsible for otolith agenesis in these mutants, we used two complementary approaches. The first approach was Whole Genome Sequencing of the *csr* mutant genome to identify regions of high homology. This indeed was difficult as the *csr* background strain was heavily inbred, resulting in multiple peaks of high homology. Since we demonstrated *csr* and *nco* are genetically-linked, we sought to further clarify the responsible locus using a second method (i.e. RNA-seq of the *nco* transcriptome) for comparison. This result pinpointed a region of high homology near the end of the 24^th^ chromosome. While deciphering potentially deleterious mutations within that region, we focused on *pks1* following evidence that it is responsible for otolith nucleation in Japanese medaka [25]. While these species are evolutionarily divergent, the shared phenotype between medaka and our mutants suggested that the role of *pks1* is conserved. As a result, we chose to use medaka *pks1* nucleic acid to rescue otolith formation in *csr, nco* and *vns* mutants. Similarities can also be drawn with other zebrafish mutants such as *keinstein*, which has diffused expression of Starmaker within the otocyst and exhibits similar circling swimming behaviors [41, 42]. Furthermore, *keinstein* may be another *pks1* allele due to its predicted chromosomal location [43].

While WT medaka *pks1* rescues otolith biomineralization in *csr* and *nco*, differences in penetrance of exogenous ions on otolith formation suggested the nature of each mutation is fundamentally different. This was confirmed by Sanger sequencing that *nco* has a premature stop codon while *csr* likely makes a defective protein that may be stabilized by exogenous ions. This defective protein may be the explanation for the differences in Mitotracker localization in *csr*. Due to its surface stain expression, we hypothesize that Mitotracker was localized to mitochondria-rich ionocytes [44]. Ionocytes have previously been implicated in otolith formation as mutations in *gcm2*, which is responsible for ionocyte maturation, leads to otolith agenesis [20, 45]. We hypothesize that the endolymph in *csr* and *nco* mutants has the necessary components for otolith nucleation [2] but lack a trigger factor produced by *pks1*. The absence of *pks1* does not visibly appear to affect hair cell development that are required for otolith nucleation either [9]. It has been previously suggested that apolipoprotein could potentially bind polyketide synthase [4, 25]. Given our RNA-seq analysis of *nco*, we see no significant change in any apolipoprotein expression. Publicly-available *in situ* data does not support Apolipoprotein expression within the inner ear [35]. Additionally, IF of *csr* and *nco* embryos demonstrated that expression of a critical otoconial seeding protein, Oc90, within the otocyst is not sufficient for otolith biomineralization in the presence of the otolithic membrane.

One caveat is that the penetrance of otolith formation is influenced by the genetic background of zebrafish. When treated with the small molecule 31N3, WT embryos in the AB/EKW background fail to develop otoliths [10]. However, 31N3 fails to inhibit otolith formation in the TL and TU strains, suggesting that there are potential genetic modifiers that influence otolith nucleation in these backgrounds. While the *csr* mutation (A911P) leads to otolith agenesis in the AB background, homozygosity at the locus is compatible with proper development in the AB/TL background (data not shown). This suggests *csr* may be a hypomorphic allele and the AB background can overcome the loss of Pks1 function with enhanced ion flux. Ironically, the mutant phenotype was lost when *csr* was outcrossed to the WIK background. It was only until *csr* was backcrossed to the AB background that the mutants were recovered. Altogether, we suggest that the AB background heavily influences the penetrance of otolith formation.

While *pks1* likely acts as an enzyme whose expression is enriched in adult mechanosensory hair cells [32], its product is required for otolith nucleation in zebrafish. However, the molecular function of *pks1* remains unknown. Using *nco* RNA-seq data, we performed an Ingenuity Pathway Analysis, which identified eNOS and Endothelin-1 signaling as the most up- and down-regulated pathways, respectively. eNOS signaling could be impacted by *pks1* metabolites such as iromycin, which has been shown to inhibit this pathway [46]. Both eNOS and Endothelin-1 have been implicated in inner ear development and function. Notably, it has been demonstrated that these pathways are inversely related in sensorineural hearing loss [47]. An example of this is Waardenburg syndrome, caused by mutations in endothelins, which cause abnormal pigmentation and sensorineural hearing loss [48]. During early development, Endothelin-1 mRNA turns on during the critical period of otolith nucleation [35, 49] and is detected in the otic vesicle at 24 hpf [50]. Endothelin-1 and its receptor (*ednraa*) are both enriched in adult zebrafish inner ear support cells [32]. Additionally, Endothelin-1 has been identified as a potential modifier of osteoblast function to increase bone mineralization [51]. Furthermore, Endothelin-1 has been implicated with the FOS-family of genes (*fosab, fosb*, and *fosl1a*) and *socs3a*, which are all differentially expressed in *nco* at 24 hpf. These genes are all part of a regulatory network during hypergravity-mediated bone formation [52]. Furthermore, the presence of osteoblast-associated proteins within teleost otoliths suggest a common mechanism between bone mineralization and otolith biomineralization [4]. Future studies will attempt to clarify the roles of Endothelin-1 and eNOS signaling pathways during biomineralization events.

## Supplementary Material

1

## Highlights

- Mutations in polyketide synthase lead to otolith agenesis in zebrafish
- Neither Otoconin-90 or Otogelin are sufficient for otolith seeding and tethering
- Endothelin-1 and eNOS signaling likely mediate otoconia biomineralization
