# Supplementary material for: Zebrafish Otolith Biomineralization Requires Polyketide Synthase
Source: Mech Dev. Author manuscript; Available in PMC 2020 Jun 1. (PMC6531356; doi:10.1016/j.mod.2019.04.001)
Supplement: 1 [file NIHMS1527800-supplement-1.docx]

Appendix A- Supplemental Material


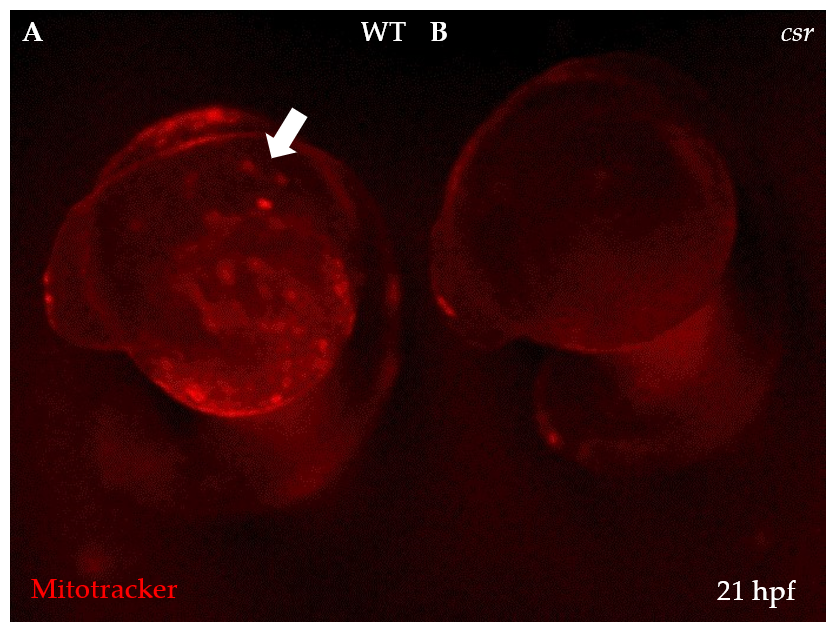


**Figure S1:** Spatial differences in mitochondrial membrane potentials. (**A**) While Mitotracker marks active mitochondria in WT, (**B**) *csr* embryos show a lack of Mitotracker expression during early development. Arrow indicates otic vesicle.


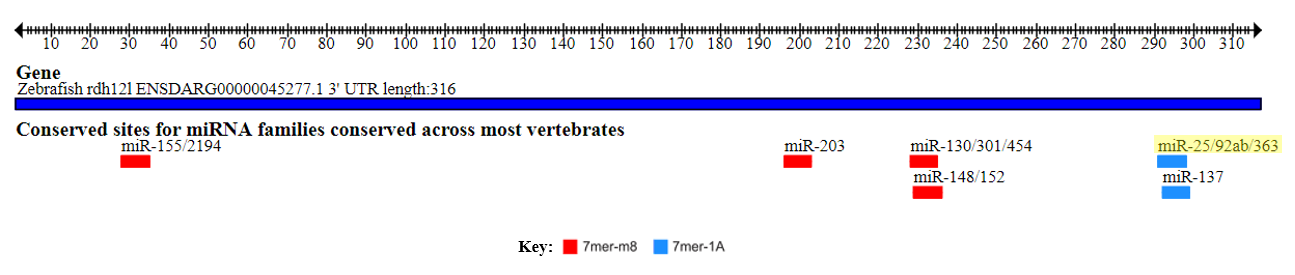


**Figure S2**: *miR-92a* binding site in the 3’ UTR of *rdh12l.* TargetScanFish 6.2 of *rhd12l* in zebrafish shows potential microRNA binding sites including *miR-92a,* which is the most down-regulated gene in *nco* embryos at 24 hpf.


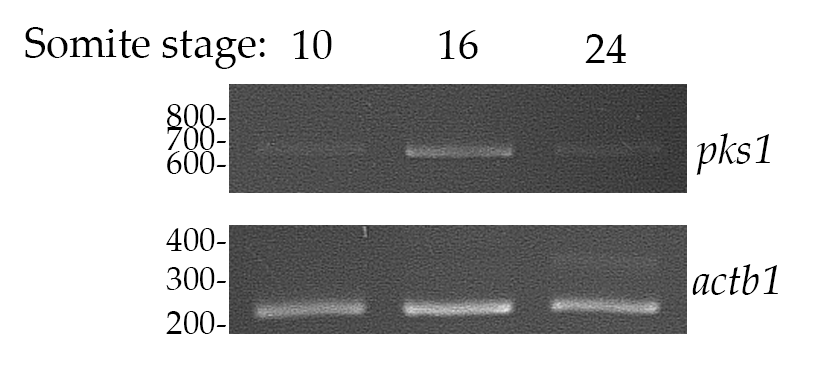


**Figure S3:** pks1 expression during early inner ear development. Using primers from distinct exons and total RNA from several developmental stages, RT-PCR was used to asses expression of pks1 and actb1 as a control. Amplification of pks1 cDNA is predicted to produce a 602bp product, while genomic contamination should produce a 789bp product. actb1 cDNA should yield a 249bp product, while gDNA should yield a 349bp product.

**Table S1.** Frequency of WT and mutant phenotypes for uninjected and injected *csr*, *nco*, and *vns* embryos.

**Table S2.** Differential expression of *pks1* in adult zebrafish hair and support cells.
